# Evaluation of Ambient Voice Technology in the National Health Service in England: Protocol for a Phase 2 Study

**DOI:** 10.2196/105261

**Published:** 2026-10-05

**Authors:** Stephen Morris, Jenny Shand, Theo Georghiou, Kevin Herbert, Rachel Lawrence, Raj Mehta, Pei Li Ng, Lucina Rolewicz, Holly Elphinstone, Holly Walton

**Affiliations:** 1 Department of Public Health and Primary Care, University of Cambridge, East Forvie Building, Cambridge, CB2 0SR, United Kingdom, 44 1223 330300; 2 Department of Clinical Educational and Health Psychology, University College London, London, United Kingdom; 3 Research and Policy, Nuffield Trust, London, United Kingdom; 4 Research Department of Behavioural Science and Health, University College London, London, United Kingdom; 5 Public Contributor, London, United Kingdom

**Keywords:** ambient voice technology, AI scribes, clinical documentation, National Health Service, NHS, mixed methods, health economics, quasi-experiment, qualitative research, artificial intelligence

## Abstract

**Background:**

Ambient voice technology (AVT) uses conversational AI to record and organize clinical consultations in real time. It is being adopted quickly across the National Health Service (NHS). However, evidence about its effects on productivity, costs, or staff experience across different health care settings is limited. Phase 1 of this research program developed a taxonomy, logic model, and outcome framework for evaluating AVT. Phase 2 will carry out a multisite, mixed methods evaluation of AVT in 4 NHS trusts. These include mental health outpatient services, acute hospital outpatient clinics, and accident and emergency departments.

**Objective:**

This study aims to explore the real-world impact of AVT on productivity, costs, and staff experience across NHS adult services.

**Methods:**

The study includes three parts: (1) a quantitative quasi-experimental analysis of routine NHS data to estimate the impact of AVT on documentation time, clinician activity, and service outcomes; (2) a comprehensive health economic evaluation comprising cost-consequence analysis, cost-benefit analysis, and budget impact modeling; and (3) interviews with up to 36 staff from 3 services (mental health outpatient, acute hospital outpatient, and accident and emergency) to explore their experience of using AVT, their views on its impact, and what helps and gets in the way of its use. Sites will be chosen to include different care settings, organizational environments, and AVT products. Quantitative and economic analyses will use NHS electronic health records and national datasets to understand changes over time and measure the impact of AVT. Interview data will be analyzed using thematic analysis and rapid assessment procedures to identify key themes. All study findings will be combined to give a clear view of AVT and its impact.

**Results:**

Data collection is expected to begin in August 2026 and conclude by January 2027. Publication of results is anticipated in February 2027.

**Conclusions:**

This evaluation will provide real-world evidence on the productivity, economic, and experiential impacts of AVT in the NHS. Outputs will include peer-reviewed papers, a slide-deck summary for the funder, and a publicly available health economic decision-support tool to help NHS organizations decide whether they should adopt AVT.

## Introduction

### Background and Rationale

Clinical documentation accounts for a substantial proportion of clinicians’ working time, contributing to workload pressures, staff burnout, and inefficiencies in patient care [1-5]. Estimates suggest that up to 25% of a clinician’s day may be spent on record-keeping and administrative tasks rather than direct patient contact [2]. Evidence also suggests that clinicians spend an average of 2 hours outside of clinical hours on documentation [6]. In parallel, health care systems are working to increase efficiency and improve productivity [5]. Ambient voice technology (AVT), which uses conversational AI to capture, structure, and document clinical encounters in real time, offers a potential solution by reducing the documentation burden, improving communication and patient-centeredness, and supporting more timely and accurate patient records [1,4,7]. Early studies suggest potential benefits for clinician-patient communication and care quality; however, the evidence for these effects is largely based on staff perceptions rather than direct patient experience measurement, and this evaluation does not assess patient experience directly (see the *Patient Experience* section).

Phase 1 of this evaluation established the conceptual and empirical foundations for a robust evaluation [8]. It included a scoping review of the evidence base [9], development of a taxonomy of AVT products, a national market map of National Health Service (NHS) adoption, and setting-specific logic models and outcome measurement frameworks. Phase 1 findings highlighted rapid but heterogeneous uptake, substantial variation in functionality and integration, and a limited evidence base. Importantly, while early evaluations frequently report perceived time savings from reduced documentation time and improved experience, there is limited evidence on how saved time is redeployed in practice, whether system-level productivity gains are realized, or what the economic consequences are at the organizational scale.

Phase 2 is a multisite, mixed methods implementation study combining quantitative and economic evaluation of AVT use in real-world NHS settings, with qualitative work to understand factors influencing implementation and outcomes, staff experiences, and views on perceived impact. This evaluation therefore focuses on whether AVT generates measurable time savings, how those savings translate into service and economic benefits, and what factors shape implementation and staff experience. It does not include a direct evaluation of AVT documentation quality or patient experience data; these are identified as priorities for future research.

### Aims and Objectives

The aim of this program of work is to conduct an independent evaluation to assess the real-world quantitative, economic, and the factors influencing implementation and outcomes of AVT in adult services in mental health outpatients, hospital outpatients, and accident and emergency settings in the NHS in England. The evaluation will focus on (1) how AVT has been implemented and used in practice, (2) the extent to which AVT generates measurable time savings in documentation and workflow, (3) how any time savings are used at the clinician and service level, (4) the downstream impacts on patient pathways and service performance, (5) the costs and budgetary implications of AVT implementation, and (6) staff experiences of using AVT for documenting patient consultations and perceptions of impact, including its impact on productivity, and the factors that have shaped implementation.

### Research Questions

The research questions are as follows:

1. How has AVT been implemented and used for documenting clinical patient consultations in mental health outpatient services, acute hospital outpatient services, and accident and emergency settings?
2. What is the impact of AVT implementation on clinical documentation processes and clinician time use in mental health outpatient services, acute hospital outpatient services, and accident and emergency settings?
3. Whether and how any time saved is used in practice, including effects on service capacity, quality of care, and workforce experience?
4. What are the wider impacts of AVT implementation on patient pathways and service performance, including timeliness of care, throughput, reattendance, and documentation quality?
5. What are the full costs of implementing and maintaining AVT at site level, and what are the short- and medium-term budgetary and economic consequences for NHS organizations?
6. How do staff experience the use of AVT for documenting clinical patient consultations?
7. What factors (barriers and facilitators) influence staff experiences and the perceived impact of AVT, including unintended consequences?
8. How do impacts, costs, and implementation experiences vary across care settings, organizational contexts, and stages of deployment maturity; to what degree has AVT been implemented as intended; and what explanatory mechanisms might account for this variation?

Collectively, these questions are designed to provide the evidence base needed by NHS England and integrated care boards to make informed decisions about AVT commissioning and scale-up. Findings will also identify what evidence is missing before national roll-out can be recommended with confidence.

### Patient Experience

Patients and members of the public are not included as research participants in this phase 2 study. The evaluation is intentionally focused on system-level productivity, economic consequences, and workforce experience, using routine service data and staff perspectives to address urgent policy and commissioning questions about AVT adoption within the NHS. Phase 1 of this program confirmed that decision-makers require robust evidence on productivity and value realization to inform investments from national productivity funding.

The absence of patient experience data should not be interpreted as a lack of commitment to understanding patients’ needs. Rather, this phase is designed to establish the foundational evidence on whether, and under what conditions, AVT delivers measurable operational and economic benefits at organizational scale. We explicitly acknowledge that excluding patient perspectives is a limitation of the evaluation, particularly given that improvements in care quality or clinician-patient interaction may represent important, though less readily quantifiable, forms of value.

Meaningful recruitment of patients to assess experience in a rapid, multisite evaluation is challenging. Superficial or underpowered patient participation was therefore considered a greater risk than exclusion at this stage. Patient and public contributors will instead provide input to study design, interpretation of findings, and output dissemination, helping to ensure relevance and transparency (see the *Patient and Public Involvement and Engagement* section). Any subsequent work moving into detailed implementation evaluation or longer-term outcomes should include appropriate patient, carer, and members of the public as research participants, alongside staff and service data.

## Methods

### Study Design

Phase 2 will comprise a multisite, rapid mixed methods evaluation of AVT implementation in 4 NHS trusts in England. The study will integrate quantitative quasi-experimental analysis, health economic evaluation, and qualitative methods to generate robust evidence on productivity, pathway, and budgetary impacts, alongside staff experiences, views on perceived impact, and influencing factors (ie, barriers and facilitators).

The evaluation will be undertaken in 2 acute trusts using AVT in both hospital outpatient services and accident and emergency (A&E) departments and 2 mental health trusts using AVT in outpatient services. The design is informed by the logic model and outcome frameworks developed in phase 1 [8] and will test the hypothesized causal pathway whereby AVT use reduces documentation burden, alters clinician time allocation, and may subsequently affect service capacity, patient pathways, and organizational costs. Setting-specific logic models will be developed for each of the following settings: mental health outpatient services, acute hospital outpatient services, and A&E departments to guide data collection and analysis.

Quantitative and economic components will form the primary analytical focus, with qualitative research undertaken in parallel to support interpretation and understand staff experiences and perceived impact across sites and settings.

### Setting and Site Selection

Sites will be purposively selected to ensure variation in care setting (acute and mental health); AVT product type, as defined by the AVT taxonomy and integration model, ensuring a range of companies and a focus on functionality rather than any specific product; stage of deployment maturity and breadth of deployment (although all selected sites are expected to have deployed AVT); and organizational context and digital maturity, to capture variation in EPR systems, data infrastructure, and readiness for AVT integration.

In the case of acute hospital outpatient services and mental health outpatient services, sites will be preferred where AVT is used in multiple different outpatient clinics so that between-clinic variation can be explored. Trusts will be preferred where AVT is used in at least two outpatient specialties, and where possible, there will be some between-trust overlap in the specialties selected. As part of the descriptive work in phase 2, we will contextualize the 4 study sites against the broader national landscape of NHS AVT adoption, drawing on the market map and supplier registry data established in phase 1, to indicate the representativeness of the sample.

Eligibility criteria include active AVT use in adult services, use of AVT products registered with Medicines and Healthcare products Regulatory Agency and included on the NHS England supplier registry [10], and the ability to provide relevant routine data and implementation information. Given the likely future use of AVT in terms of integration with electronic patient records (EPRs), to future-proof the analysis, a desirable criterion is sites that use AVT products that are fully integrated into the electronic health record (EHR), meaning that AVT-generated notes are either automatically appended to the patient record as structured or free text or that consultation-level flags indicating AVT use are recorded in the EPR. The presence of such flags is particularly important for the quantitative analysis, as it enables consultation-level comparison of AVT and non-AVT encounters.

### Quantitative Analysis

#### Design

The quantitative work packages will have 2 main elements. First, descriptive data will be sought and analyzed to document the scale of AVT use in each setting and site and to understand the implementation in the context of the broader service. AVT exposure will be defined at the most granular level available at each site. Where consultation-level AVT flags are available, consultations using AVT will be compared with non-AVT consultations within the same service, adjusting for clinician, clinic, time period, type, and mode of consultation (eg, follow-up and in person), and patient case-mix. Patient case-mix will be defined using available demographic and diagnostic variables from routine data (eg, age, sex, ethnic group, deprivation score, available diagnosis or acuity information, and referral source). The specific variables available will vary by site and will be prespecified in the statistical analysis plan following feasibility assessments. Where only aggregate implementation dates or adoption metrics are available, exposure will be defined at the clinician, clinic, specialty, department, or site level. The analysis plan will prespecify the exposure definition for each site and assess the risk of exposure misclassification. Data will also be accessed on patient and process outcomes that might be potentially impacted by AVT use and the availability of comparator information will be assessed. Second, this information will be used to design analyses to test the evidence of any association between AVT adoption and consultation time measures and other service and system outcomes. Given that randomized allocation is not feasible in this context, quasi-experimental methods will be used. Depending on the data available, we will use the most appropriate method or methods, potentially including pre-post comparisons within sites, interrupted time series analysis, within-site comparator analyses (eg, services such as outpatient clinics for other specialties not using AVT or preimplementation periods), and multivariable regression modeling to adjust for case-mix.

The choice of analytic method for each site will be determined following a formal data feasibility assessment and prespecified in a statistical analysis plan (SAP) to be reviewed by the Project Advisory Group before any analysis commences. As a rule, interrupted time series analysis will require a minimum of 12 months of preimplementation data and 6 months of postimplementation data, and pre-post comparisons will require a minimum of 6 months pre- and post implementation. Where these thresholds cannot be met, the study will default to descriptive and dose-response analyses. Comparator clinics or services will be selected from within the same trust where possible, prioritizing those with similar patient case-mix and activity volumes but without AVT exposure, with selections documented and justified in the SAP. Concurrent service changes will be recorded through site-level contextual data collection throughout the study period and, where identified, reported transparently and controlled for in regression models or used to define sensitivity analysis periods. Where AVT has been rolled out in phases within a site, the phased implementation timeline will be used to define exposure and, where sufficient variation exists, to support dose-response analyses.

The analyses will use data routinely collected by each service. Each analysis will be at a level appropriate to the specific service and to the data, including comparator data, available (eg, clinician level, clinic level, or specialty level). Similar data sources have been used in prior evaluations of digital health innovations, and at least one trust implementing AVT has demonstrated that they are able to interrogate their EPR system to extract consultation-level data (including with AVT use recorded). All sites will undergo a formal data feasibility assessment before being confirmed, during which the availability of preimplementation data and the adequacy of the historical time series will be established. Sites will only be recruited where the existence of relevant routine data can be demonstrated.

If the scale of the implementation allows, local data analysis may be supplemented with analyses using national hospital datasets (Hospital Episode Statistics and the Emergency Care Data Set). In the absence of data on AVT use linked to individual consultations, national datasets such as these may be used to design dose-response analyses to compare rates of AVT use over time with specific outcomes. The latter would either also be derivable from national data or otherwise supplied at an aggregated level from the trusts. These analyses would need day-by-day or week-by-week counts of AVT use by specialty. We may also use these national datasets to contextualize site-level information.

In general, each of the 3 types of setting will have different data available and will require a specific quantitative evaluation approach. Within each setting, however, we will aim to implement the same analysis across each of the 2 sites to strengthen the quality of the evidence.

#### Data Sources

Data will be obtained from EHR systems, other local routine administrative datasets (eg, activity and performance data), AVT product usage metrics, and national hospital datasets (Hospital Episode Statistics and the Emergency Care Data Set).

Data sharing agreements will be established with each participating trust, and we will develop a Data Protection Impact Assessment. Data will be pseudonymized or aggregated prior to transfer and will be analyzed in a secure research environment.

#### Potential Data Outcomes

The primary outcome of interest is documentation time (measured via EPR time stamps), alongside secondary outcomes, which may include consultation duration, total EHR activity time, proportion of consultations closed on the same day, proportion of letters sent on the same day, time to consultation closure, time to patient letter having been sent, out-of-hours EHR time, and clinical activity volume.

The outcomes may be constructed either per consultation (eg, documentation time per consultation) or per day or shift (eg, number of consultations per day).

Availability of each outcome variable will be established through site-level feasibility assessments conducted prior to finalizing the SAP, and outcomes will only be included where data quality and completeness are sufficient. The following data items will be accessed:

1. AVT product use: number of AVT-supported consultations per day, clinician, clinic, specialty, or A&E shift (extent of use); history of AVT use (longitudinal information); descriptive data on demographics of patients for whose consultations AVT has been used; and AVT product usage information (type of AVT used, session counts, duration of use, number of edits made, template use, and number of clinicians using the product).
2. Contextual information within the same services: number of consultations per day, clinician, clinic, specialty, and A&E shift; history of number of consultations; and descriptive data on demographics of patients.
3. Potential impacts related to EPR use: time spent in EPR as recorded by EPR system activity logs, where available; out-of-hours time spent in EPR; time stamps related to consultation and documentation events (consultation start and end time, consultation mode [eg, in person], onward plans confirmed [eg, follow-up, referral, or discharge], consultation closed, letter complete and sent, subsequent edits of notes, and in A&E: time to complete the first entry on EPR and time from a clinician being assigned to patient to decision made about patient disposal [eg, discharged, bed booked, or transport booked]); whether AVT use is recorded against individual consultation data; and consultation data completeness and data detail (eg, number of primary and secondary diagnoses recorded for the consultation; noting that completeness is a proxy for documentation quality and does not capture clinical accuracy of AVT-generated content, which is out of scope).
4. Potential impacts related to wider service and system: number of attendances or A&E patients per day or clinician shift; number of first outpatient attendances, referrals made, people discharged, and number of days from referral to assessment or first attendance.

#### Analysis

An SAP will be prepared and reviewed by the Project Advisory Group before the analysis commences and will confirm the primary, secondary, and exploratory outcomes. Analyses will proceed in four stages:

1. Descriptive analysis of baseline service characteristics and AVT uptake patterns (including analysis of inequalities in AVT use by patient demographics);
2. Time series analysis examining pre- and postimplementation trends in consultation time measures (alternatively, depending on the data available, a dose-response analysis);
3. Regression modeling adjusting for case-mix, time trends, organizational and clinical factors (eg, EPR system type, AVT product used, stage of deployment maturity, specialty, and concurrent service change events), and patient demographic characteristics to enable examination of inequalities in outcomes; and
4. Sensitivity analyses exploring heterogeneity by site, setting, and maturity of deployment (eg, by stratifying or repeating the primary analyses separately for each setting).

The unit of analysis will ultimately be dependent on the data available (whether, eg, AVT use is recorded for specific consultations) and may be at the individual consultation level or at an aggregated level. Where possible, analyses undertaken during the earlier months of the analysis time period (see timeline) will be updated with additional data shortly before the final reporting stage.

Results will be presented with appropriate measures of statistical confidence. Where causal inference is limited by data constraints, findings will be interpreted cautiously.

Direct analysis of AVT summary outputs and resulting patient notes (and other correspondence) for errors or potential errors will be out of scope for this evaluation. However, any local analysis of errors related to AVT use will be sought.

Where the qualitative workstream identifies unanticipated issues with measurable quantitative markers (eg, evidence of unintended workflow changes or differential AVT use by staff group), these will be explored in the quantitative data where feasible, subject to data availability. Any such exploratory analyses will be clearly distinguished from prespecified analyses in the SAP.

Key risks to the quantitative workstream include limited availability of pre-implementation data, absence of consultation-level AVT flags, incomplete EPR time stamp data, delayed or inconsistent AVT deployment, and product changes during the study period. Contingency plans include restricting to aggregate-level analysis where consultation-level data are unavailable, supplementing EPR data with locally held administrative datasets, and using interrupted time series designs that are robust to gradual implementation. A formal risk register will be reviewed at each Project Advisory Group meeting.

### Health Economic Evaluation

The economic evaluation will adopt an NHS and Personal Social Services perspective as the primary analytic viewpoint, supplemented by a broader societal perspective where relevant data permit (eg, capturing patient time costs or productivity losses). This is consistent with National Institute for Health and Care Excellence reference case guidance and the HM Treasury Green Book framework for public sector appraisal [11]. The primary analytic framework will be a cost-consequence analysis, presenting costs and a disaggregated profile of outcomes side by side to allow decision-makers to form their own judgments about the relative importance of different effects. This will be accompanied by a cost-benefit analysis in which outcomes that can be expressed in monetary terms are valued and combined with costs to yield an overall net monetary benefit, a benefit-cost ratio, and a net present value.

A budget impact analysis will further model the short- to medium-term financial implications of AVT adoption at site, system, and potential national scale. Where data permit, a cost-effectiveness analysis framing the marginal cost per unit of additional clinical activity will also be explored. All costs and benefits after the first year will be discounted at 3.5% per annum in line with HM Treasury Green Book recommendations (op cit.). Costs and outcomes will be reported in current UK prices and inflated where necessary using the NHS Cost Inflation Index or the PSSRU (Personal Social Services Research Unit) Unit Costs of Health and Social Care. Costs will be identified and measured through structured site-level resource questionnaires, financial data on licensing and subscription fees, staff time associated with implementation and training, IT integration and governance resource use, and ongoing maintenance and support costs.

The cost components will be as follows:

1. *Technology acquisition costs*, for example, software licenses and subscription fees to AVT suppliers, hardware (microphones, tablets, and integration devices), procurement and tendering costs (including staff time on procurement activities), information governance setup costs (data processing agreements and information asset registration), and regulatory compliance costs
2. *Implementation and integration costs*, for example, technical integration with EPRs, IT project management and deployment costs across sites, clinical safety and data governance activities, cybersecurity and secure hosting
3. *Training and change management costs,* for example, training clinicians and support staff
4. *Operating and maintenance costs*, for example, annual maintenance or subscription renewals for AVT platforms, upgrades and feature updates, technical support, quality assurance, and auditing of AI-generated notes
5. *Service delivery costs*, for example, changes in consultation length, increased patient throughput, reduced administrative time, and potential staff-time cost offsets, such as less need for medical secretaries or manual transcription
6. *Costs associated with unintended consequences of AVT use*, including clinician time spent reviewing or correcting inaccurate or incomplete AVT-generated notes and any downstream clinical or administrative costs arising from documentation errors
7. *Broader system costs and cost offsets*, for example, reduced errors in documentation, potentially avoiding downstream costs associated with miscoding or clinical mistakes, and lower burnout-related costs

Outcomes will be derived from the quantitative analysis workstream. Identified effects will be monetized using established unit cost sources. The primary approach to valuing clinician and staff time will use the PSSRU Unit Costs of Health and Social Care, supplemented by NHS Agenda for Change pay scales and employer on-costs (approximately 30% on-cost for National Insurance and pension contributions). Time savings will be converted to a monetary value by multiplying estimated hours saved per clinician by the appropriate hourly cost. A key methodological consideration is the extent to which freed time translates into additional productive activity (“value realization”), as opposed to being absorbed into existing workloads. A range of assumptions about value realization will be tested in sensitivity analyses, from full conversion (all time saved generating additional consultations) to zero (all time absorbed as slack), with a central estimate informed by qualitative data from site interviews. Where freed time translates into additional clinical activity, the value realized will be estimated by valuing the time savings directly rather than by applying NHS tariff to additional activity because the consultation cost already incorporates staff time and double-counting would occur if both were summed. The analysis plan will clearly distinguish between valuing time savings (the primary approach) and valuing additional activity generated (an alternative scenario tested in sensitivity analyses). Where effects cannot be monetized—for example, improvements in documentation quality, staff well-being, or patient experience—they will be reported descriptively within the cost-consequence framework.

A budget impact and return on investment (ROI) model will examine the trajectory of cumulative costs and benefits over a 3-year time horizon, with extrapolation to 5 years in a secondary analysis. This will allow estimation of the payback period—the point at which cumulative monetized benefits exceed total implementation costs—and a net present value and benefit-cost ratio for each site and setting. Analyses will be conducted at site level and, using typical adoption pattern assumptions derived from the evaluation, at hypothetical national scale to inform commissioning decisions. An openly accessible ROI decision-support tool will be produced as a deliverable from this workstream, enabling NHS organizations to apply the framework to their own settings using local cost and activity data.

Uncertainty in the economic analysis will be characterized through (1) deterministic one-way sensitivity analysis, varying each key parameter (adoption rate, value realization rate, license cost, and staff grade mix) across plausible ranges informed by phase 1 evidence and the published literature; (2) scenario analysis, testing predefined pessimistic, central, and optimistic cases; and (3) probabilistic sensitivity analysis, where sufficient data are available, propagating parameter distributions through Monte Carlo simulation to yield cost and benefit CIs. Threshold analyses will identify the minimum time saving per consultation or adoption rate required for AVT to be cost-neutral or cost-saving from an NHS perspective. Sensitivity and scenario findings will be reported alongside base-case results in the final report and associated publications.

### Qualitative Analysis

#### Aims

The qualitative workstream primarily addresses research questions 1, 6, 7, and 8 but also contributes to answering research questions 2 to 5 by providing contextual explanations of quantitative findings (eg, explaining how and why time savings are or are not realized in practice and identifying the mechanisms underlying variation across sites). The qualitative workstream will explore (1) staff experiences of using AVT for documenting clinical patient consultations, (2) the perceived impact of AVT for this purpose, and (3) factors influencing experiences and perceptions of impact (barriers and facilitators). This evaluation will not include interviews with patients, as this is not within the scope of the work. However, during interviews with staff members, we will cover points relating to communication with patients and the perceived impact of AVT on patient care.

#### Design

We will use a qualitative design using semistructured interviews. We will aim to draw on relevant theories and concepts during design and analysis (eg, relating to behavior change [12], risk work [13,14], and staff roles relating to embedding new technologies within an organization [15]). Qualitative topic guide development and data collection will be informed by early quantitative descriptive findings on patterns of AVT use and service characteristics. Where descriptive analyses identify potential inequalities in AVT uptake or use, these will be incorporated as prompts in interview topic guides.

#### Sampling and Recruitment

Within the qualitative workstream, we will conduct interviews with staff in 3 of the 4 sites included in the quantitative and health economic workstreams. This will enable us to explore the implementation of and staff experience with AVT in-depth. Across these 3 trusts, we will conduct up to 36 interviews in total (Table 1). Within each trust, we will explore one of the 3 settings (eg, site 1: hospital outpatients, site 2: A&E, and site 3: outpatient mental health). We will conduct up to 12 interviews with staff per setting (Table 1). Where settings cover multiple specialties and/or departments (ie, hospital outpatients), we will select one clinic where AVT is being used based on the maturity of deployment and informed by ongoing discussions with sites during recruitment.

**Table 1.** Overview of qualitative data collection.

Trust^a^	Setting	Number of interviews	Examples of staff roles across all sites or settings (and number of interviews across roles)^b^		
			Interviews with staff who have wider oversight of AVT^c^ implementation and use (where relevant or in place)	Interviews with staff in roles working directly with AVT in practice	Interviews with staff in roles working with or receiving AVT outputs
A	Hospital outpatients (one specialty and/or department)	Up to 12	Up to 6 interviews with, for example, clinical and/or operational leads, governance or safety officers, project managers, suppliers, and data and IT teams	Up to 24 interviews with, for example, physicians, consultants, nurses, psychologists or mental health professionals, allied health professionals, trainees or students. One of the interviews in each site will include a follow-up interview to explore ongoing perceived impact	Up to 6 interviews with, for example, receptionist and/or administrative staff, patient navigation and/or coordination roles, health care assistants, and technicians
B	A&E^d^	Up to 12			
C	Mental health outpatients	Up to 12			

a For the qualitative work, we will sample 3 of the 4 trusts selected to participate in this evaluation.

b Examples presented in this table are not exhaustive and will depend on local setup across the different settings. During site recruitment, we will discuss recruitment of staff and work with leads to identify suitable roles to interview during the evaluation. This will help to ensure that we interview all important staff groups.

c AVT: ambient voice technology.

d A&E: accident and emergency.

We will aim to purposively recruit staff members working in different roles across settings, including staff working directly with AVT in practice during consultations and developing notes, staff members in a role which works with the receipt of AVT outputs, and staff with wider oversight of AVT implementation and use (Table 1). Inclusion and exclusion criteria for qualitative interview participants are presented in Textbox 1.

Textbox 1. **Inclusion and exclusion criteria for qualitative interview participants.**

Inclusion criteria

- Staff working in or with the participating trusts who are involved in the oversight or implementation of ambient voice technology (AVT), directly use AVT for documenting clinical patient consultations, and/or receive AVT outputs
- Staff aged ≥18 years
- Staff who are English speaking or able to participate in an interview
- Staff who can provide informed consent to participate

Exclusion criteria

- Anyone aged <18 years who cannot provide informed consent to participate
- Staff not working in the participating trusts

#### Recruitment and Consent Processes

Qualitative researchers will work with site leads to identify staff members who meet the specified eligibility criteria and would be appropriate to interview. Potential participants will be contacted via email to invite them to participate. Site leads and eligible staff members may also share study details with their networks to support recruitment and invite anyone who is interested to contact the researchers. Participation in the study will be voluntary. All potential interviewees will be sent a participant information sheet and consent form via email after expressing an interest in taking part. Participants will then have at least 48 hours to review the information sheet and consider whether to participate. If participants are happy to take part, they will be asked to provide consent prior to the interview (either written or audio-recorded verbal consent). Participants will be free to withdraw at any time during the interview and can withdraw their data up to 2 weeks after the date of the interview.

#### Data Collection

Participants will be contacted to arrange a convenient time for a remote interview via phone or Microsoft Teams. Interviews will last approximately 30 to 60 minutes, be semistructured, be audio recorded on an encrypted Dictaphone (subject to consent), be transcribed verbatim by a professional transcription service, be anonymized, and be kept in compliance with the General Data Protection Regulation (GDPR) and Data Protection Act (2018) [16]. Interviews will be scheduled to take place during regular working hours, as staff are not being compensated for their time.

#### Topic Guides

Topic guides have been iteratively developed, guided by phase 1 findings and scoping [8] and relevant theories (eg, capability, opportunity, motivation–behavior model used as prompts to explore factors influencing delivery and implementation of AVT [12] and nonadoption, abandonment, scale-up, spread, and sustainability [NASSS] framework to explore the embedding of new technologies within an organization [15]).

Interviews will broadly cover the following topics: knowledge about AVT and any previous experiences of use, drivers to implementation and anticipated impact, the AVT tool and its integration into the clinical pathway and the trust’s IT architecture (including EPR integration, data flows, and technical dependencies), implementation of AVT into practice, patient consent and communication, experiences of using AVT (where relevant), perceived impact of AVT, any unintended consequences, views on future use, and the factors influencing implementation, experiences, and perceived impact. Some of the questions will be tailored to draw out issues relevant to the specific settings. Topic guides will include prompts to draw out examples in specific settings. All participants will be asked to provide some sociodemographic information (sharing this information will be voluntary). This will include job role, length of time in post, and length of time using AVT in practice.

#### Analysis

To analyze qualitative data, we will use a medium Q thematic analysis approach [17], which combines an inductive approach with the use of a coding framework [18]. Data collection and analysis will be conducted in parallel, using rapid assessment procedure (RAP) sheets [19] guided by topic guide questions (with flexibility to add categories inductively during the research process). Qualitative data will be analyzed by named researchers in the rapid service evaluation team (RSET). Notes will be taken in real time by qualitative researchers during the interviews and inputted onto RAP sheets following each interview. RAP sheet notes will be used to develop initial themes and subthemes, which will be used to develop a coding framework. Following the rapid analysis, an in-depth analysis will be undertaken, and researchers will apply the coding framework to all transcripts, to produce final themes and subthemes. There will be flexibility to add codes inductively to contribute to theme development during the analysis. Findings will be integrated with quantitative and economic results during the synthesis stage to provide a coherent account of AVT use and impact.

### Integration

Quantitative, economic, and qualitative findings will be integrated in three phases of integration: (1) iterative sharing of emerging findings across workstreams during data collection, (2) use of qualitative findings to explain and contextualize quantitative results during analysis, and (3) formal triangulation of all 3 workstreams. This synthesis will allow explanation of heterogeneity across sites; identification of necessary conditions for productivity gains; differentiation between perceived and measurable impacts; development of refined outcome monitoring recommendations; and understanding of the factors that influence implementation, use, and outcomes of AVT across settings and contexts.

A particular focus of the triangulation process will be to understand if there are any time savings associated with AVT, and if so, how those time savings are used in practice.

### Ethical Considerations

The Health Research Authority decision tool indicates that this study is classified as a service evaluation and therefore does not require formal Health Research Authority ethics review. Ethical approval will be sought from the University College London (UCL) Research Ethics Committee prior to commencement of data collection (status: application submitted; decision pending). A Data Protection Impact Assessment has been completed and registered in line with UCL’s Data Protection Policy (reference Z6364106/2026/07/69 clinical research & DPIA-101). Data governance arrangements will comply with the UK GDPR, the Data Protection Act 2018, and NHS information governance standards. Data shared between participating trusts and the study team will be aggregated or pseudonymized wherever possible and transferred via approved secure mechanisms. Data sharing agreements will be established, where required, between UCL and each participating trust prior to any data transfer.

All interview participants in the qualitative workstream will provide informed consent (see *Recruitment and Consent Processes section*). Data will be stored and analyzed in secure environments.

### Data Management

The study is compliant with the requirements of the GDPR (2016/679) and the UK Data Protection Act (2018). All investigators and study site staff will comply with the requirements of the GDPR (2016/679) with regard to the collection, storage, processing, and disclosure of personal information and will uphold the Act’s core principles. UCL, the Nuffield Trust, and the University of Cambridge are joint data controllers and processors.

### Patient and Public Involvement and Engagement

The study team includes a public contributor and the National Institute for Health and Care Research (NIHR) RSET Patient and Public Involvement and Engagement (PPIE) panel. The panel currently comprises 8 members representing a range of backgrounds and relevant patient experiences. Our public contributor attends project team meetings and has contributed to the development of the study design, research questions, and this protocol. The RSET PPIE panel will be invited to comment on data collection materials, emerging findings, and dissemination outputs. All PPIE contributors will be compensated in line with NIHR’s payment guidance [20].

Prior to the commencement of data collection, we will hold a PPIE workshop to review the study design and qualitative topic guides. Further PPIE engagement is planned at key milestones, including the review of interim findings and dissemination materials. Feedback from PPIE contributors will be recorded on a PPIE feedback log to ensure transparency and enable the impact of PPIE input on study procedures and decision-making to be tracked throughout the project. Given that this evaluation does not directly involve patients as participants, PPIE will provide an important mechanism for incorporating patient and public perspectives throughout the study. This will help ensure that the evaluation remains focused on issues of relevance, acceptability, and real-world importance to patients and the public and supporting the development of accessible and meaningful dissemination outputs. While PPIE provides an important mechanism for incorporating the patient perspective, it is not a substitute for empirical patient experience data. The limitations of excluding patients as research participants are discussed fully in the *Patient Experience* section and in the *Strengths and Limitations* section.

### Equity, Diversity, and Inclusion

Equity, diversity, and inclusion (EDI) is a cross-cutting consideration for all workstreams. We applied or will apply the NIHR RSET EDI project-specific assessment tool [21] at three stages: (1) during protocol development, (2) during data collection and analysis, and (3) following completion of data collection. The assessment covers EDI considerations across recruitment, data collection, analysis, and dissemination. Where patient demographic data (age, sex, ethnicity, and deprivation) are available from routine sources, analyses will include descriptive breakdowns of AVT use and outcomes by these characteristics to identify inequalities in access to or impact of AVT. Availability of these variables will be established through site-level data feasibility assessments, and the analysis plan will specify which demographic variables will be included for each site. Within the qualitative workstream, topic guides will include prompts to explore staff views on whether and how AVT affects different patient groups differently and whether any barriers to equitable use exist from a staff perspective. Within the health economic workstream, the budget impact analysis will consider implications for organizations serving more deprived populations. The EDI assessment will also consider the potential for differential AVT performance across patient groups, including patients with accents or dialects; those with speech impairments, cognitive impairments, neurodivergent conditions, or sensory impairments; and those with limited English. As AVT outputs are not directly reviewed in this evaluation, inequities in transcription accuracy or summarization quality may not be directly observable. Where data permit, we will seek to record and report on which patients are not offered AVT, decline its use, or are considered unsuitable by clinicians to provide a partial account of equity of access. Dissemination outputs will be developed in accessible formats with input from PPIE contributors.

## Results

The study protocol was approved by the funder in May 2026. Data collection is scheduled to begin in August 2026 and is expected to be completed by January 2027. Final results are expected to be published by February 2027.

## Discussion

### Summary

This protocol describes a rapid, multisite, mixed methods evaluation of AVT in the NHS in England. Phase 2 builds directly on the conceptual and empirical foundations established in phase 1, which produced a taxonomy, logic models, and outcome frameworks to guide robust evaluation [8]. The study integrates quantitative quasi-experimental analysis and a comprehensive health economic evaluation (across 4 trusts) and qualitative interviews within 3 of these NHS trusts. The evaluation will take place in different settings, including mental health outpatient, acute hospital outpatient, and accident and emergency settings. The evaluation will assess the extent to which AVT generates measurable time savings, how those savings are used in practice, and the downstream implications for service capacity, patient pathways, and organizational costs. It will also examine staff experiences of using AVT, the factors that facilitate or impede its use, and how impacts vary across settings and contexts.

### Strengths and Limitations

The strengths of this evaluation include its mixed methods design, which enables triangulation of quantitative, economic, and qualitative evidence. The study builds on phase 1 logic models and outcome frameworks [8], providing a theoretically grounded basis for data collection and analysis. The inclusion of multiple sites across different care settings—mental health outpatient, acute outpatient, and accident and emergency—will allow setting-specific and cross-setting comparisons, enhancing the generalizability of findings. The health economic component is more comprehensive than most existing AVT evaluations, explicitly modeling value realization and conducting sensitivity analyses to address parameter uncertainty. The openly accessible ROI tool will translate study findings into a practical resource for NHS commissioners and trusts. The use of rapid methods, that is, RAP and parallel data collection and analysis, is well suited to the pace of AVT adoption in the NHS.

There are, however, important limitations. As a rapid evaluation, the study is constrained in its ability to detect longer-term impacts on patient outcomes or workforce retention, which may take years to materialize. The 4-site design, while enabling depth of analysis within each setting, limits the breadth of AVT products, organizational contexts, and implementation approaches that can be captured. Given the rapid and highly heterogeneous nature of AVT adoption across the NHS, especially with numerous products, EPR integration approaches, and deployment models, the findings should be interpreted as indicative of what is achievable in a rapid evaluation and as hypothesis generating rather than as definitive national evidence. Purposive site selection mitigates this to some extent, but generalization should be approached cautiously. Quasi-experimental methods, while appropriate in the absence of randomization, are subject to confounding from simultaneous changes in care delivery. Data availability will vary across sites and settings, and the extent to which routine NHS data can capture the relevant outcomes of interest (particularly documentation time) will not be fully known until feasibility assessments are completed. The qualitative component covers only a subset of staff in each site and will not capture the full range of views across all roles or patient perspectives. Economic estimates will depend heavily on local assumptions about value realization, and the results should be interpreted in light of the sensitivity analyses. Finally, as a team using rapid service evaluation operating across 4 sites, this study makes methodological trade-offs in the interests of timeliness and policy relevance. These include a limited sample size relative to the diversity of NHS AVT adoption, reliance on available routine data rather than purpose-designed data collection, and a focus on staff perspectives rather than patient experience. These constraints affect the generalizability of findings, and the results should be interpreted accordingly alongside the specific contexts of the study sites. Throughout all outputs, the study will clearly distinguish between effects measured directly from routine data, effects reported by staff as perceived impacts, and inferences about patient experience. Claims about communication, care quality, or patient-centeredness will be attributed specifically to staff perceptions unless directly evidenced by objective data.

### Implications

This evaluation has significant implications for NHS policy and practice. AVT is being rapidly adopted across the NHS without robust independent evidence on its productivity impact, cost-effectiveness, or how time savings translate into service benefits. This study will provide the first comprehensive, multisite economic and quantitative evaluation of AVT in the NHS, generating evidence that NHS England, integrated care boards, and individual trusts can use to inform commissioning and procurement decisions. The qualitative findings will explore the conditions under which AVT is most effective and acceptable and identify factors that influence effective implementation, supporting more targeted implementation. The health economic decision-support tool will be freely available to NHS organizations, enabling local adaptation of the evaluation framework to guide investment decisions in the absence of locally collected data. The findings will also inform the growing international evidence base on AI-enabled clinical documentation.

### Future Research

This rapid evaluation is necessarily bounded in scope and time horizon. Future research should address several gaps that this study cannot fully resolve. First, a longer-term evaluation of the impact of AVT on patient outcomes (including referral accuracy, treatment timeliness, and reattendance rates) would require data linkage over a longer follow-up period than is feasible here. Second, a formal cost-effectiveness analysis using quality-adjusted life years or other standardized health utility measures would require richer patient-level outcome data. Third, as AVT products evolve rapidly, their functionality (particularly EPR integration, multimodal capabilities, and AI-generated coding) will change substantially; ongoing evaluation and horizon-scanning will be needed. Fourth, evaluation of patient experiences of AVT-enabled consultations, including consent, trust, and communication, warrants dedicated study with patient participants. Fifth, comparative evaluation across different AVT vendors and products would help commissioners distinguish between the generic effects of AVT as a category of technology and the specific features of individual products. Finally, evaluation in primary care settings, which were out of scope for this study, represents an important priority, given the volume of consultations and the current pace of GP adoption. In addition to the specific future research priorities identified, this evaluation may itself highlight unanticipated gaps or questions (eg, emerging evidence of harms, unexpected benefits, or population subgroups requiring closer attention). These will be flagged in the final outputs as additional priorities for future investigation.

### Conclusions

This protocol describes phase 2 of the NIHR RSET rapid evaluation of AVT in the NHS. The study will generate timely, multisite, mixed methods evidence on the productivity, economic, and experiential impacts of AVT across mental health outpatient, acute outpatient, and accident and emergency settings. By integrating quantitative, health economic, and qualitative methods and by grounding the evaluation in phase 1 logic models and outcome frameworks, it will produce comprehensive and policy-relevant findings. The study will be conducted by an experienced, multidisciplinary team and overseen by an independent project advisory group. Outputs will include peer-reviewed publications, a final NIHR report, and a publicly available economic decision-support tool, contributing to the evidence base needed to guide responsible, effective, and equitable adoption of AI-enabled clinical documentation technologies in the NHS.

## Supplementary material

10.2196/105261 Peer Review Report 1 Peer reviewer record 1.

10.2196/105261 Peer Review Report 2 Peer reviewer record 2.

## Abbreviation

A&E: accident and emergency
AVT: ambient voice technology
EDI: equity, diversity, and inclusion
EHR: electronic health record
EPR: electronic patient record
GAIDeT: Generative AI Delegation Taxonomy
GDPR: General Data Protection Regulation
Gen-AI: generative AI
NASSS: nonadoption, abandonment, scale-up, spread, and sustainability
NHS: National Health Service
NIHR: National Institute for Health and Care Research
PPIE: Patient and Public Involvement and Engagement
PSSRU: Personal Social Services Research Unit
RAP: rapid assessment procedure
ROI: return on investment
RSET: rapid service evaluation team
SAP: statistical analysis plan
UCL: University College London
