# Supplementary material for: Evaluation of Ambient Voice Technology in the National Health Service in England: Protocol for a Phase 2 Study
Source: JMIR Res Protoc. 2026 Oct 5;15:e105261. doi: 10.2196/105261 (PMC13637992; doi:10.2196/105261)
Supplement: Supplementary file 1 [file resprot-v15-e105261-s001.pdf]

RSET Light-touch Peer Review Guide

Project Title: Evaluation of Ambient Voice Technology in the NHS in England - Phase 2 Study Protocol

Peer Reviewer Name: Dr Judith Harrison

Overall comment: Strong and policy-relevant rapid evaluation.

Could you sharpen scope and inference, especially around patient experience, documentation safety /clinical quality?

Background: [Rapid Service Evaluation Team (RSET)] is a collaboration between researchers from UCL, the Nuffield Trust, and University of Cambridge. We are conducting rapid evaluations of health and care service innovations, in close partnership with those who deliver, manage and use these services, and will produce timely findings of national relevance and immediate use to decision-makers. In order to issue timely results to influence change, we will make use of innovative, cutting edge approaches to data collection, analysis, and sharing lessons from our research. Our goal is to undertake two evaluations per year for five years. The protocol attached here outlines one such evaluation that will be undertaken by the RSET researchers.

Instructions: Please review the project protocol and provide a response where indicated. If you would like to elaborate, please provide any further comments in the 'Comments' column or on a separate page securely attached. Please do not edit or comment directly in the protocol.

Question	Response	Comments (if applicable)
1. Does the research/evaluation address an important issue or problem for the organisation and delivery of health care services or health policy?	☒ Yes<br>☐ No<br>☐ Unable to answer	Highly relevant/urgent for NHS service delivery. AVT may reduce documentation burden and release clinician time, but it also raises important questions about safety, accountability, consent, trust, documentation quality, workflow and inequalities. The protocol would be stronger if it stated more clearly how findings will inform national adoption decisions, and what evidence would still be needed before wider scale-up?
2. Do the research/evaluation aims, questions and/or hypotheses build on and address gaps in existing knowledge?	☒ Yes<br>☐ No<br>☐ Unable to answer	protocol addresses an important gap whether AVT produces measurable benefits in real NHS settings, beyond perceived time saving or vendor claims.<br><br>But gaps could be framed more sharply? not only whether AVT saves time, but whether time is redeployed into service benefit, whether documentation remains clinically safe, whether errors are detected and corrected, and whether impacts vary by setting, patient group, clinician role or product maturity – and what can't be assessed in this study should be acknowledged as limitation/for future work
3. Are the design/methods proposed appropriate? Are they robust enough to address the research/evaluation questions?	☒ Yes<br>☐ No<br>☐ Unable to answer	The mixed-methods design is appropriate for a rapid evaluation focused on productivity, economics and staff experience.<br>But routine data may not capture documentation quality/clinical usefulness/safety.<br><br>Think we should specify primary, secondary vs exploratory outcomes. how documentation quality will be assessed given direct review of AVT outputs is out of scope. how will AVT-related errors, omissions or near misses will be captured? negative cases, non-use, workarounds and unintended consequences – how will these be actively sought?
4. Are the research/evaluation plans realistic and achievable in the time frame proposed, given any problems which may arise?	☒ Yes<br>☐ No<br>☐ Unable to answer	Broadly realistic, but dependent on timely access to usable routine data, clear AVT exposure measures and sufficient uptake. Main risks are delayed deployment, inconsistent use, lack of consultation-level AVT flags, incomplete EPR time data (and I think this is could be significant), variation between sites, weak comparator groups.<br><br>Add a short contingency plan for low uptake, missing data, product changes etc?
5. Has the research/evaluation meaningfully involved patients, service users, or the public in its design and/or planned delivery?	☐ Yes<br>☐ No<br>☒ Unable to answer	PPIE plans are appropriate within the chosen scope, and the public contributor/PPIE panel are welcome.<br>I think excluding patients as participants is a major limitation. given affects on privacy, trust, disclosure, rapport, consent, perceived clinician attention etc. Staff perceptions and PPIE input are not substitutes for empirical patient experience data, and PPIE in the abstract – not embedded in the experience – may not get at the issues.<br><br>make this limitation prominent and identify patient/carer experience as a priority for follow-on evaluation.

6.	Have equity, diversity, and inclusion considerations been appropriately addressed in the research/evaluation design and population focus?	☒ Yes<br>☐ No<br>☐ Unable to answer	EDI is appropriately identified as cross-cutting. descriptive analysis by age, sex, ethnicity and deprivation is useful<br><br>The protocol could go further on accent/dialect/language/speech impairment/cognitive impairment, neurodivergence/mental health symptoms/sensory impairment, communication complexity. Because patients are not participants and AVT outputs are not directly reviewed, inequities in transcription accuracy, summarisation quality or acceptability may be missed.<br><br>Could you report who is not offered AVT, declines it, or is judged unsuitable where possible?
7.	Does the work have the potential to positively impact health and/or social care delivery or policy?	☒ Yes<br>☐ No<br>☐ Unable to answer	Yes. The study could strongly inform NHS policy, procurement and implementation, particularly through the economic workstream / ROI tool.<br><br>impact will be greatest if outputs clearly separate measurable productivity gains, perceived benefits, implementation requirements, unresolved safety questions, evidence gaps.<br><br>avoid overstating conclusions about patient benefit, clinical safety or documentation quality if these are not directly assessed
8.	Will the dissemination plan help engagement with relevant stakeholders and promote timely feedback of the research findings?	☒ Yes<br>☐ No<br>☐ Unable to answer	dissemination plan is strong and seems appropriately targeted<br><br>I suggest adding a concise implementation and assurance checklist for NHS organisations considering AVT, covering consent, transparency, clinical safety, documentation review, information governance, staff training, workflow integration, equity monitoring, post-deployment audit?<br><br>Public-facing outputs should clearly explain what the study did and did not evaluate, especially regarding patient experience/accuracy of AI-generated documentation
9.	Are there any changes/improvements you would like to propose at this stage?	☒ Yes<br>☐ No<br>☐ Unable to answer	See comments above
