# Supplementary material for: Evaluation of Ambient Voice Technology in the National Health Service in England: Protocol for a Phase 2 Study
Source: JMIR Res Protoc. 2026 Oct 5;15:e105261. doi: 10.2196/105261 (PMC13637992; doi:10.2196/105261)
Supplement: Supplementary file 2 [file resprot-v15-e105261-s002.pdf]

RSET Light-touch Peer Review Guide

Project Title: Evaluation of Ambient Voice Technology in the NHS In England: Phase 2 Study Protocol

Peer Reviewer Name:

Background: Rapid Service Evaluation Team (RSET) is a collaboration between researchers from UCL, the Nuffield Trust, and University of Cambridge. We are conducting rapid evaluations of health and care service innovations, in close partnership with those who deliver, manage and use these services, and will produce timely findings of national relevance and immediate use to decision-makers. In order to issue timely results to influence change, we will make use of innovative, cutting edge approaches to data collection, analysis, and sharing lessons from our research.

Instructions: Please review the project protocol and provide a response where indicated. If you would like to elaborate, please provide any further comments in the 'Comments' column or on a separate page securely attached. Please do not edit or comment directly in the protocol.

Question	Response	Comments (if applicable)
1. Does the research/evaluation address an important issue or problem for the organisation and delivery of health care services or health policy?	X Yes<br>☐ No<br>☐ Unable to answer	
2. Do the research/evaluation aims, questions and/or hypotheses build on and address gaps in existing knowledge?	X Yes<br>☐ No<br>☐ Unable to answer	
3. Are the design/methods proposed appropriate? Are they robust enough to address the research/evaluation questions?	☐ Yes<br>☐ No<br>X Unable to answer	Depends on points made in response to Q4 below.
4. Are the research/evaluation plans realistic and achievable in the time frame proposed, given any problems which may arise?	☐ Yes<br>☐ No<br>X Unable to answer	"The analyses will use data routinely collected by each service."<br>And<br>"Where consultation-level AVT flags are available, consultations using AVT will be compared with non-AVT consultations within the same service, adjusting for clinician, clinic, time period and patient case-mix"<br>The quant analysis would fall at the first hurdle if these data, including for the pre-AVT period,

		are not available. There is a lot of text built on the assumption that they are available but can you give some reason(s) why you think they are and that it will be possible to get them in the timescale available? Please explain and if possible cite sources or references to studies that have used the sources you are intending to use.<br><br>“Data will also be accessed on patient and process outcomes that might be potentially impacted by AVT use and the availability of comparator information will be assessed.”<br>Which patient and process outcomes? You name some data sources but it is unclear they will actually have the outcomes data you need and be able to make it available to you within the timescale of the project. Need to clarify.<br><br>“If the scale of the implementation allows, local data analysis may be supplemented with analyses using national hospital datasets (Hospital Episode Statistics (HES), and the Emergency Care Dataset (ECDS)). ”<br>Unclear how you might be able to use high level aggregate data for this study. Explain.<br><br>“, each of the three settings will have different data available and will require a specific quantitative evaluation approach. Within each setting, however, the same analysis will be implemented across each of the three sites”<br><br>?I thought there were 4 sites (2 acute, 2 mental health) and in effect >3 settings depending on which, and how many different,

		OP specialties you select at 2 the acute hospitals.<br><br>"Activity gains will be valued using NHS national tariff or reference costs."<br>But those costs are costs without using AVT. If an OP appointment's cost changes because of AVT you need to allow for that. Better not to value the activity gains but rather to value the time savings. To add together the values of both would be double counting.<br><br>You state that "The qualitative workstream aims to answer research questions 5 and 6"<br>But you need qual analysis to help answer Qs1,2 and 3 as well.<br><br>On p9 you say "Trusts will be preferred where AVT is used in at least two outpatient specialities, and where possible there will be some between-Trust overlap in the specialities selected."<br>But then in Table 1 on p17 you say you will only interview for one specialty. So why the earlier reference to 2 or more specialties? Confusing.
5. Has the research/evaluation meaningfully involved patients, service users, or the public in its design and/or planned delivery?	X Yes<br>☐ No<br>☐ Unable to answer	
6. Have equity, diversity, and inclusion considerations been appropriately addressed in the research/evaluation design and population focus?	☐ Yes<br>☐ No<br>X Unable to answer	P21 says:<br>"We will apply the NIHR RSET EDI project-specific assessment tool[20] at three stages: (i) during protocol development"<br><br>You have developed the protocol. So no longer future

		tense. Did you use that tool and what did that reveal?<br><br>"Within the quantitative workstream, all analyses will include descriptive breakdowns of AVT use by patient demographic characteristics (age, sex, ethnicity, deprivation) to identify inequalities in access to or impact of AVT."<br>But will those demographic data be available? Doubtful, surely. See earlier comments about giving reason to believe these data exist, or being less certain (viz "will include") in your wording of the protocol. What will you do if the data are not available – should recognise that possibility in the protocol unless you are certain that they are available. So wording would be better along the lines "if these data are available we will use them".<br><br>"topic guides will include prompts to explore whether and how AVT affects different patient groups differently"<br>Make clear you mean explore with staff their views on whether … You are not interviewing patients, you say.
7. Does the work have the potential to positively impact health and/or social care delivery or policy?	X Yes<br>☐ No<br>☐ Unable to answer	
8. Will the dissemination plan help engagement with relevant stakeholders and promote timely feedback of the research findings?	X Yes<br>☐ No<br>☐ Unable to answer	
9. Are there any changes/improvements you would like to propose at this stage?	X Yes<br>☐ No<br>☐ Unable to answer	See earlier comments. Plus I would expect to see an assessment of project risks, their likelihood and their impact if they occur (not least unavailable quant data), and the mitigating actions that would be

		taken. This could be in the form of a table.
